# Hydrogel-Forming Algae Polysaccharides: From Seaweed to Biomedical Applications

**DOI:** 10.1021/acs.biomac.0c01406

**Published:** 2021-02-12

**Authors:** Marco Beaumont, Remy Tran, Grace Vera, Dennis Niedrist, Aurelie Rousset, Ronan Pierre, V. Prasad Shastri, Aurelien Forget

**Affiliations:** † Queensland University of Technology, Brisbane, Australia; ‡ Institute for Macromolecular Chemistry, University of Freiburg, Freiburg, Germany; § Centre d’Étude et de Valorisation des Algues, Pleubian, France; ∥ Centre for Biological Signalling Studies, University of Freiburg, Frieburg, Germany

## Abstract

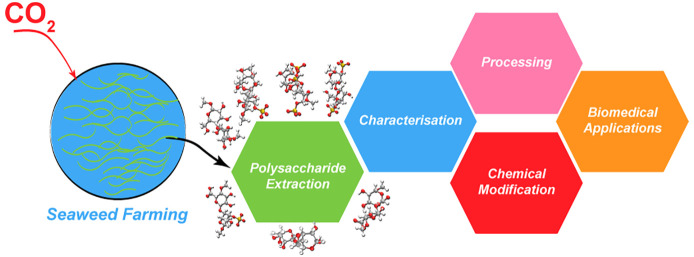

With the increasing growth of the algae industry and the development of algae biorefinery, there is a growing need for high-value applications of algae-extracted biopolymers. The utilization of such biopolymers in the biomedical field can be considered as one of the most attractive applications but is challenging to implement. Historically, polysaccharides extracted from seaweed have been used for a long time in biomedical research, for example, agarose gels for electrophoresis and bacterial culture. To overcome the current challenges in polysaccharides and help further the development of high-added-value applications, an overview of the entire polysaccharide journey from seaweed to biomedical applications is needed. This encompasses algae culture, extraction, chemistry, characterization, processing, and an understanding of the interactions of soft matter with living organisms. In this review, we present algae polysaccharides that intrinsically form hydrogels: alginate, carrageenan, ulvan, starch, agarose, porphyran, and (nano)cellulose and classify these by their gelation mechanisms. The focus of this review further lays on the culture and extraction strategies to obtain pure polysaccharides, their structure-properties relationships, the current advances in chemical backbone modifications, and how these modifications can be used to tune the polysaccharide properties. The available techniques to characterize each organization scale of a polysaccharide hydrogel are presented, and the impact on their interactions with biological systems is discussed. Finally, a perspective of the anticipated development of the whole field and how the further utilization of hydrogel-forming polysaccharides extracted from algae can revolutionize the current algae industry are suggested.

## Introduction

Food, water, minerals, and the movement of commodities and natural resources through trade routes provide us with a multitude of resources that are vital for life on earth. The exploitation of these resources or ocean economy is estimated to reach an industrial scale of USD 3 trillion by 2030 (USD = United States dollars).^1^ While the oceans cover over 71% of the earth surface,^2^ over 80% of the ocean is unmapped, unobserved, and unexplored.^3^ In addition to the fauna that is consumed for food, the plant-like algae growing in the oceans are now a focus of interest for diverse applications including food, biofuel, or as a carbon dioxide reservoir.^4^ On land, soil plants have been used for food production but also as materials for construction, and the extraction of cellulose has led the establishment of thriving textile and paper industries. While the ocean on our planet covers a considerably larger area than land mass, the utilization and exploitation of oceanic plant-like resources at an industrial scale are still in infancy. With the available area that oceans offer for culture and with developing knowledge on algae, there is an untapped potential for the emergence of an industry based on materials extracted from algae.

Algae, which can be unicellular or multicellular, unlike plants, lack a vascular system and are classified as eukaryotic organisms. They play a vital role in regulating the carbon dioxide and oxygen in our atmosphere, by functioning as a carbon sink and releasing oxygen as part of photosynthesis. To be used as materials, marine algae polysaccharides must exhibit interesting properties. One particular property of several algae-extracted polysaccharides is their capacity to form hydrogels, making them suitable for several commercial applications. Hydrogels are defined as a three-dimensional (3D) macromolecular network that are highly swollen in water but do not dissolve.^5^ Beyond the application of algae-extracted hydrogel-forming polysaccharides as gelling agents or rheology modifiers in food applications,^6^ some of these materials have found applications in the high-added-value field of biotechnology.^7^

In the laboratory, hydrogel-forming marine polysaccharides such as agarose have been used since the 1970s as a support medium in the analysis and separation of DNA strands and, since the 19th century, as microorganism culture media.^8^ The process of electrophoresis is familiar to biologists and is a routine technique in any molecular biology laboratory. The extensive use of agarose in molecular biology has led to the fundamental understanding of the electrophoresis process, optimization of the extraction and purification processes of agarose, and deep understanding of the red algae farming and the impact of geographical and seasonal variation on the quality of the extracted agarose. In cosmetic formulations, carrageenan is now used for facial masks and in topical creams.^9^ Similarly, in medical devices, alginates are used in the formulation of wound-dressing hydrogel-based pastes,^10^ and production of algal nanocellulose has recently gained more attention,^11^ as it bears great potential for biomedical applications.^12^

While these biomedical applications have placed marine algae under the limelight, only a few polysaccharides, extracted from a handful of algae, are currently used. With the increase of our understanding of marine algae and a deeper knowledge of algae farming and culture, new uses for algae products will have to be identified to valorize these agricultural advances. Beyond the obvious food application, high-added-value applications such as the biomedical uses of polysaccharides could bring opportunities for creating a flourishing industry.

To achieve this, several avenues are possible and are discussed in this review article. One is to find new uses for the hydrogels currently derived from algae. This requires a deep understanding of their physicochemical properties. Concomitantly, current polysaccharides could be chemically modified to introduce functional groups conferring key properties for biomedical applications. The chemical modification of polysaccharides can be achieved through either coupling of functional moieties or direct modification of the saccharides’ repeat unit. Finally, extension of the current library of seaweed-extracted hydrogel-forming polysaccharides would allow the development of a new area of applications. Such new polysaccharides could be discovered through the development of advanced extraction methods, and the discovery of new seaweed species from which yet unknown polysaccharides could be isolated.

In this article, we present the current library of hydrogel-forming polysaccharides extracted from algae, their chemical properties, and mechanisms of gelation. We further discuss characterization methods applied to polysaccharides and their resulting hydrogels. Then, we present chemical modifications that can be used to tailor the polysaccharide properties. Finally, we discuss the polysaccharide properties that are critical for their biological performance and envision future industrial developments related to hydrogel-forming polysaccharides.

## Hydrogel-Forming Seaweed-Extracted Polysaccharides

### Hydrogel Formation Mechanism

A hydrogel is defined as a 3D network formed by hydrophilic polymer chains connected by cross-linking. These chemical properties provide a hydrogel with high water-swelling capacity while being nonwater-soluble. Physically, hydrogels are characterized by a lack of flow under the cuvette inversion test, due to a much larger storage moduli than loss moduli (*G*′ ≫ *G*′′)^13^ and a linear plateau region of the storage modulus,^14^ and can hence be classified as a rheological soft solid.^15^ These properties are attractive for the biomedical field, as hydrogels can reproduce the hydration conditions of natural mammalian tissues^16,17^ and mimic some of the physical properties of the extracellular matrix composed of polysaccharides such as hyaluronic acid and protein such as collagen.^18,19^ Within the class of hydrogel-forming polymers, polysaccharides represent a prominent family of macromolecules. One of the main sources for hydrogel-forming polysaccharides is seaweed. As such, algae-extracted polysaccharides have had a tremendous impact on the field of biotechnology. A case in point is the extensive use of agarose hydrogel for DNA sorting and analysis.^8,20^ Without agarose, current advances in molecular biology would not have been possible. Beyond agarose, other polysaccharides extracted from seaweed have been identified, but only a few of them form hydrogels. It can be envisioned that these hydrogel-forming algae-extracted polysaccharides could be a major source of future materials for biomedical applications.

Algae-extracted polysaccharides form hydrogels through physical cross-linking, that is, noncovalent bonding that only relies on weak interactions such as hydrogen bonding, van der Waals forces, and electrostatic interactions leading to a reversible gel formation. Conversely, hydrogels such as poly(methacrylic acid) form cross-linking points through covalent bonding leading to irreversible gels and are classified as chemically cross-linked hydrogels. While this hydrogel class could be extended to chemically cross-linked hydrogel-forming polysaccharides induced by a cross-linking agent or chemical modification,^21,22^ as demonstrated for laminarin and fucoidan, we chose to focus strictly on polysaccharides that naturally form hydrogels.

We identified seven hydrogel-forming algae-extracted polysaccharides: alginate, λ-carrageenan, ulvan, starch, agarose, ι-carrageenan, κ-carrageenan, porphyran, and (nano)cellulose (Table 1). While polysaccharides extracted from algae are usually classified by the genus of their algae source, this can become challenging, since their properties are strongly dependent on species. Alginate is, for example, extracted from brown algae of the *Ochrophyta* phylum, encompassing ∼1500 algae species,^23^ such as *Laminaria hyperborea, Laminaria digitata*, and *Macrocystis pyrifera* with different alginate compositions.^24,25^ Besides the type of species,^25,26^ harvesting season and water quality affect the composition of alginates as well.^27−29^ Since there is a strong correlation between the polysaccharide structure and the properties of the resulting hydrogel, it is crucial to have a deep understanding of the structure–property relationship to gain some predictability in order to successfully use algae-derived polysaccharides for an industrial biomedical application. Additionally, the extraction methods and their conditions such as pH, temperature, and mechanical processes^30,31^ can induce changes in the polysaccharide composition and thus affect the resulting gel properties and commercial potential.

**Table 1:**
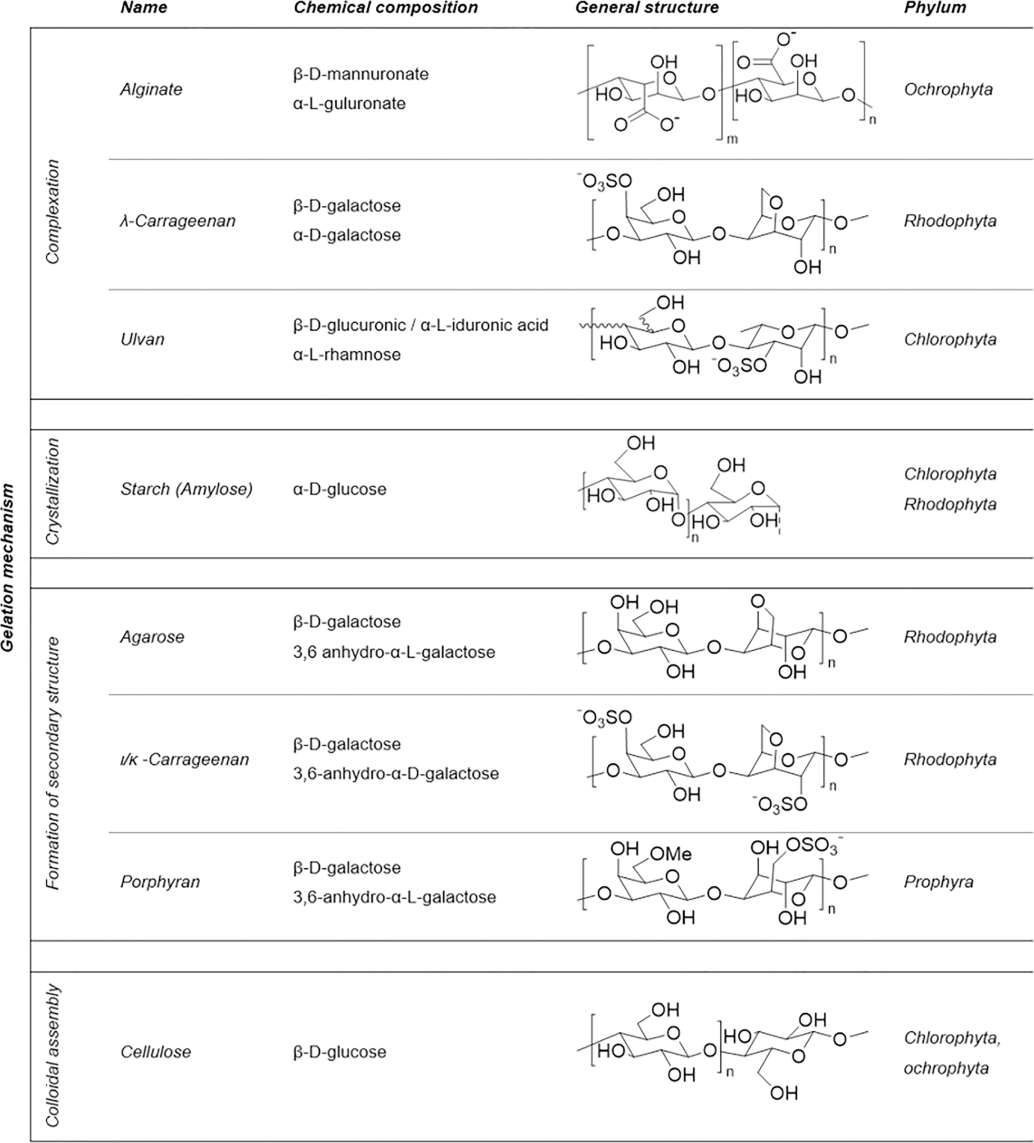
Classification of Polysaccharides According to Their Gelation Mechanisms and a List of the Main Repeating Units of These Polysaccharides, Their Chemical Structures, and Their Common Algae Sources

**Figure 1:**
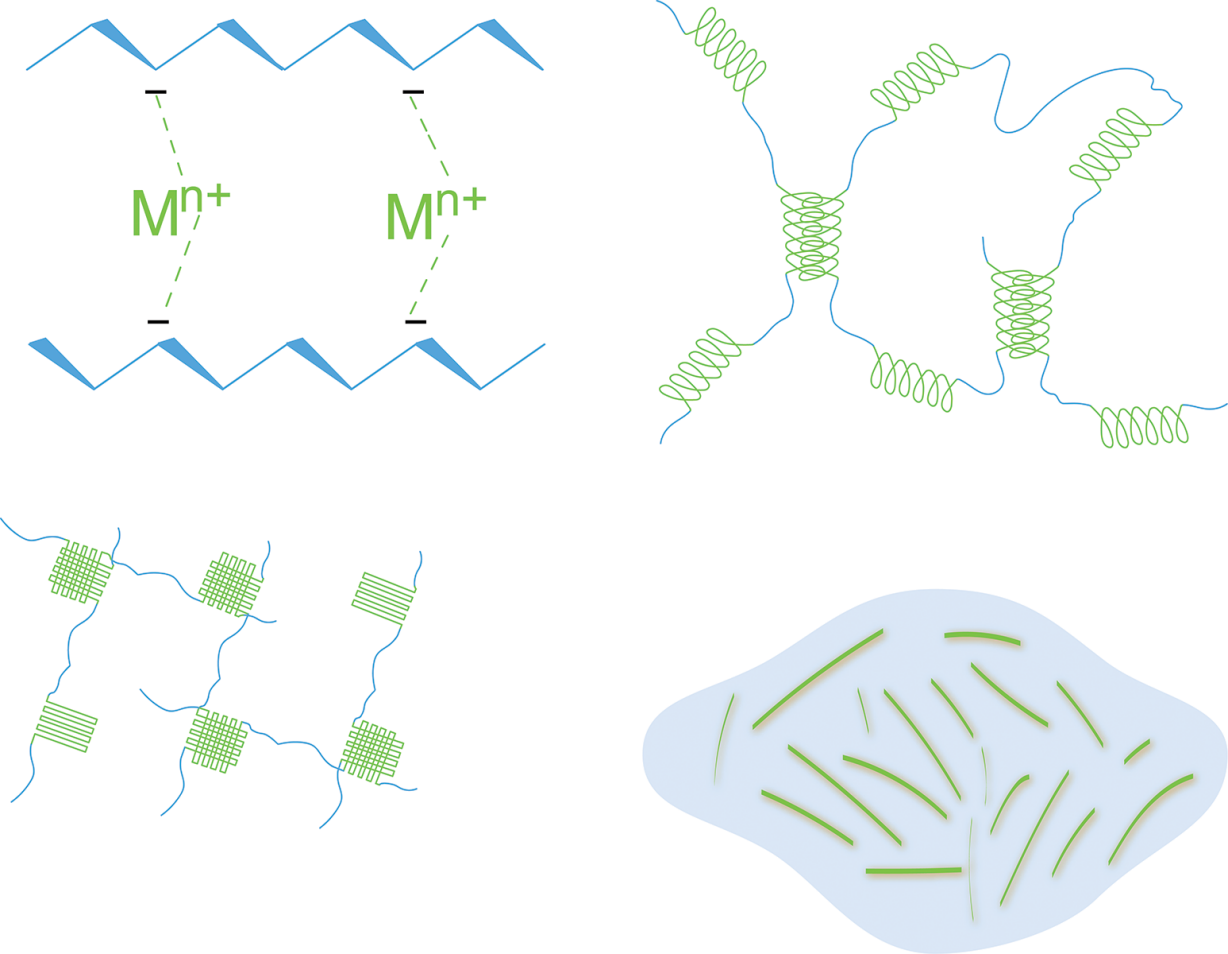
Comparison of four different main gelation mechanisms in algal polysaccharides. (A) Schematic of the complexation in ionic polysaccharides, such as alginates. (B) Aggregation of polysaccharide chains into secondary structures through a formation of double helices. (C) Formation of physical cross-links by induced crystallization in amorphous regions. (D) Gelation of colloids, such as nanocellulose, by colloidal crowding.

With a focus on industrial-scale biomedical application, we propose a classification of these hydrogel-forming polysaccharides by their mechanisms of physical cross-links. In this respect, it is important to identify the smallest subunit necessary for gelation, as it is the key element in the gelation mechanism, that is, gelator. The gelator will define the gel properties and its processing and, eventually, will determine its final applications.^32^ A deep understanding of the gelator interactions and how chemical modifications will influence them is critical to enable a tuning of the hydrogel properties and a full exploration of the polysaccharide’s potential as biomaterials. Within the seven identified hydrogel-forming algal polysaccharides, we identified four classes of gelation mechanisms, driven by either complexation, crystallization, formation of secondary structure, or colloidal assembly (Figure 1). The chemical structures of polysaccharides presented in this review and the classification into these gelation mechanisms are shown in Table 1.

### Complexation

Some polysaccharides can naturally form complexes with biomolecules such as proteins^33,34^ and lipids.^35,36^ In the case of hydrogel-forming polysaccharides, the gelation can be induced by the formation of a metal complex with the ionic groups of polysaccharides with alginates as the most common example (Figure 1A).^37^ The gelation occurs because of the formation of a binding between the polysaccharide chain and a coordination center (metals or metalloids). The latter acts as a bridge between individual macromolecular chains and thus forms a cross-linking point. In the formation of the complex, both polysaccharide and coordination center play an equal role, and this formation can be supported by the polyelectrolyte nature of the polysaccharide, through electrostatic interactions leading to further associations.^38^

***Alginate*** is one of the most studied polysaccharides, which gels via an ionic complexation. The polysaccharide structure varies greatly depending on the seaweed growth environment, leading to different polysaccharide compositions. In addition to the culture environment, the polysaccharide composition is dependent on the extracted algae tissue, as it can be extracted from the whole frond or either the algae blade or stipe. Alginate is composed of two saccharide units, β-d-mannuronic acid (M) and α-l-guluronic acid (G) arranged in sequences of M- and G-block regions and randomly inserted M and G units (MG-blocks).^39,40^ It is now well-described that the G blocks determine the stiffness and the M random regions contribute to the flexibility of the resulting polysaccharide.^41^ An algae, which is highly exposed to waves, requires a high stiffness to resist the wave’s action and will finally produce more guluronate. Hence the season^42,43^ and culture location have a huge influence on the chemical structure of polysaccharides.^44^ The difference in polysaccharide composition of the respective algae tissue can be explained similarly; the stipe that mechanically supports the algae requires a higher stiffness than the blade and, hence, is composed of polysaccharides with a higher number of G-blocks.^25,44,45^ In addition to these factors, the algae species is of course important to consider,^25,46,47^ as well as the protocol of extraction.^48^ A combination of all these factors influences the resulting polysaccharide composition and thereby their gelation and final hydrogel properties.

Once extracted, alginate gels in the presence of divalent cations such as Ca^2+^, according to the egg-box model.^37^ The divalent cations interact majorly with the carboxylate groups of the G-blocks^49,50^ (while the M-blocks have a way lower affinity) through electrostatic interactions leading to a network formation. The gelation was often seen as solely occurring through the G-blocks; however, studies from Donati et al.^51^ related the importance of the alternating MG sequences by proving the formation of mixed junctions between G- and MG-blocks through nuclear magnetic resonance (NMR). The M/G ratio is defined by the ratio of M to G units. Because of the high affinity of divalent cation toward the G-blocks, the gel properties will greatly depend on the M/G ratio and the G-block length. An alginate with a higher G content and a low M/G ratio will therefore produce a stiffer gel with higher gel strength than an alginate with a high M/G ratio.^24,52^ As the egg-box gelation requires a divalent cation, the specificity of the cation and its concentration will have an impact on the gel properties.^53^ Depending on the cation nature, the minimal concentration required for gel formation, selectivity coefficient, and mechanical properties considerably vary.^49,53^ For instance, it has been shown that Ca^2+^ exhibits stronger interactions with the alginate than Mg^2+^, and hence, lower amounts of Ca^2+^ are required to form strong hydrogels.^49,50,54^

***λ-Carrageenan*** is a linear polysaccharide composed of 1,3-linked β-d- and 1,4-linked α-d-galactose substituted with three sulfate groups per disaccharide units, and thus, in the group of selected polysaccharides, it has the highest sulfate content. λ-Carrageenan has a similar gelation mechanism to alginate. However, it is usually only described as a thickening agent unable to form hydrogels. But, a gelation mechanism based on a trivalent cation complexation was reported by Running et al.^55^ and Cao et al.^56^ The latter confirmed the specific interaction between λ-carrageenan and trivalent cations such as Fe^3+^ and Al^3+^, whereas Cr^3+^ did not cause gelation.

The high sulfate content of λ-carrageenan is of significant importance, as it can lead to antioxidant or anticoagulant properties, a key feature for its consideration in biomedical applications.^57,58^ However, factors such as species,^59^ seasons,^60^ growth conditions,^61^ and extraction processes^62,63^ are known to influence the composition. These factors have been reported to also influence the sulfate content and substitution pattern in ι- and κ-carrageenan and thus may also be of influence in λ-carrageenan. Since these chemical characteristics are key for the biological properties, the development of industrial extraction methods leading to a reproducible chemical structure is critical for their further development into biomaterials.

***Ulvan*** is a sulfated polysaccharide mainly composed of glucuronic acid, iduronic acid, rhamnose, xylose, mannose, glucose, and galactose.^64^ Several predominant repeating disaccharide patterns have been found, such as a β-d-glucuronic acid 1,4-linked with α-l-rhamnose-3-sulfate and an α-l-iduraonic acid 1,4-linked to α-l-rhamnose-3-sulfate.^65,66^ Similar to the other introduced polysaccharides, the structure and composition of ulvan was reported to considerably vary across algae species^67^ and seasons of extraction.^28,68^ Ulvan exhibits a particular gelation mechanism, which is reported to occur in the presence of boric acid and divalent cations such as Ca^2+^ leading to the formation of a thermoreversible gel.^69−72^ It is proposed that the gel occurs either through the divalent Ca^2+^ ion that acts as a bridge between the borate groups or by the cations that stabilize the coordination of borate with the hydroxyl groups of the polysaccharides.^69,70^ But, no evidence of borate-polysaccharide complexes could be found by NMR.^65^ Further investigation of the gelation mechanism has shown that factors such as the cations^67^ and boric acid concentration^70,71^ were influencing the gel properties. The metals involved in the complexation of alginate and λ-carrageenan interact differently with the ulvan polysaccharides. In ulvan gels, it was found that Cu^2+^ cations led to the formation of a stronger hydrogel than with Ca^2+^, whereas no gel formation was observed in the presence of Mg^2+^.^67^ Shedding light on the hydrogel formation mechanism of ulvan could be very beneficial and lead to interesting applications, for example, in metal coordination for the removal of metal.

### Crystallization

With respect to synthetic polymers, crystallization is a well-known process that impacts the material properties, and similar observations have been made in natural polysaccharides as well. The process of crystallization can be controlled by an application of cooling rates or anisotropic stretching of the polymer chains. In the course of crystallization, a network can form through the interconnection of crystalline regions (crystallites, spherulites) acting as junction zones between the amorphous regions.^73,74^ In synthetic polymers like polypropylene, the process is often known as a two-step mechanism involving the nucleation of crystals followed by their growth.^75,76^ However, more complex mechanisms involving spinodal decomposition^77^ or the appearance of a mesomorphic phase^78,79^ have been observed in natural polysaccharides.

***Starch*** is a polysaccharide composed of two polysaccharides, namely, amylose and amylopectin, and is primarily extracted from plants such as potato, maize, and wheat,^80^ but it also occurs in algae.^81^ Starch amylose is a linear gel-forming polysaccharide mostly composed of 1,4-linked α-d-glucose with small numbers of 1,6-linked α-d-glucose unit branches, while amylopectin is a highly branched polysaccharide composed of 1,4-linked α-d-glucose heavily interlinked with 1,6-linked units.^82^ The starch composition and ratio of amylose and amylopectin vary depending on the species and whether it is from land plants or algae, and this ratio influences the starch gelation. For instance, starch extracted from the red seaweed (Rhodophyta) called Floridean starch lacks amylose and thus does not gel.

Starch gelation is attributed to a crystallization process and occurs through gelatinization and retrogradation, which is an order–disorder transition induced by a heating and cooling cycle. Amylose forms a gel through a phase separation followed by crystallization occurring in the polymer-rich phase.^83−85^ Amylopectin contributes to the network formation through a slow retrogradation mechanism (days) that increases further the crystallinity and long-term stability.^80,85^ Because of this mechanism, the amount of amylopectin and the amylose/amylopectin ratio play an important part in the gelation.

Retrogradation is a complex process that depends on many factors such as the chain length of amylopectin and the starch phosphate content.^86−88^ As the cross-linking points are established through the crystalline regions, the concentration of the polysaccharide^85,89,90^ and the crystallization conditions such as the temperature and the cooling rate will have an impact on the crystallite morphology and thus the gel properties.^91,92^ For instance, an increase in the cooling rate has been reported to yield a softer gel, as it gives the macromolecular chains a smaller time frame to reorganize and form ordered regions.^92^

### Formation of Secondary Structure

The secondary structure of a polymer is the 3D structure adopted by the macromolecular chains. In solution, some polysaccharides can go through a coil-to-helix transition. Like DNA polymers, polysaccharides such as agarose and κ-carrageenan form double helices in solution. Once formed the helix can aggregate to create cross-linking points between the polymer chains leading to the formation of a 3D network. The aggregation of helices is driven (especially in the case of agarose^93^ or κ-carrageenan^94^) by electrostatic interpolymer chain repulsions and stabilized by weak attractive interactions. In these polysaccharide systems, the helices can be interrupted due to kinks that are induced by the irregularity in the polymer chains, which thus controls the size of the cross-linking points.^95^

***Agarose*** is one of the polysaccharides constituting agar, the other one being agaropectin, which has the same backbone as agarose but with sulfated galactose and pyruvic acid residues. The purification and extraction process of agarose is therefore an important step, as agaropectin is a nongelling polysaccharide.^26^ Agarose’s backbone is composed of β-d-galactose and 3,6-anhydro-α-l-galactose (3,6-AG) similar to the one from ι- and κ-carrageenan.^96^ Changes in the composition and structure of agarose polysaccharide such as the presence of α-l-galactose and other minor substituents (sulfate, methyl ether, pyruvic acid)^95^ are known to occur depending on the species^26,95^ and seasons.^29,97^

The composition of agarose controls the formation of secondary structures of the polysaccharide governing its gelation mechanism.^98^ It is believed that agarose gelation occurs through a phase separation mechanism, involving the formation of double helixes in the polymer backbone and aggregations of these helices into cross-linking points creating a 3D hydrogel network.^99,100^ However, the phase separation mechanism is still debated, and both spinodal decomposition^100,101^ and nucleation/growth^102^ are reported in the literature. The gelling properties are correlated with the structure of agarose, in which the equatorial hydrogens of the 3,6-AG residues force the chains into a helix.^26^ Replacing the 3,6-AG by a 6-*O*-sulfo-l-galactose interrupts the helix by a kink formation leading to a lower gel strength.^26,98^ This principle can be used to tune the mechanical properties of the hydrogel through a chemical modification. Additionally, to modulate further the gel properties, the polysaccharide concentration can be increased to induce a stronger helix aggregation resulting in a stronger gel strength.^103,104^

***ι- and κ-Carrageenan*** gelation occurs through the addition of monovalent or divalent cations to inhibit the electrostatic repulsion between the hydrogel chain due to the presence of charged groups. While ι- and κ-carrageenan have the same backbone, composed of β-d-galactose and 3,6-AG, they differ in sulfate content; ι-carrageenan possesses sulfate groups on both galactose and 3,6-AG, while κ-carrageenan features only substitution on galactose units.^105^ This difference affects the respective gelation mechanisms leading to different mechanical properties of the hydrogels, κ gels being strong and brittle while ι gels are softer.^94,106,107^ Like other algae-extracted polysaccharides, many factors such as species,^59,108^ seasons,^27,60^ growth conditions,^61^ and extraction conditions^59,109^ are influencing the 3,6-AG and sulfate content, which in turn alters the helix formation leading to different gel properties.^110^

In the presence of cations, ι- and κ-carrageenan go through a coil-to-helix transition, leading to the formation of double helices. In κ-carrageenan the helix formation is followed by further helix aggregation,^94,106,111^ but this aggregation does not occur in ι-carrageenan due to the presence of two sulfate groups inducing a stronger electrostatic chain repulsion.^112,113^ In the case of κ-carrageenan, the gelation is dependent on monovalent cations.^114^ The type of cation used to induce the gel formation will impact the mechanical properties of the hydrogel. For instance, κ-carrageenan forms a stronger gel with K^+^ than with Na^+^.^114−116^ Not only cations but also some anions such as I^–^ and SCN^–^ have been reported to bind to the helix influencing the gelation mechanism by impeding helix aggregations and gelation.^117−119^ Since the ι- and κ-carrageenan hydrogel formation is governed by their secondary structure, manipulation of this structure, for example, through the addition of ions, can have a drastic impact.

***Porphyran*** is a sulfated polysaccharide composed of alternating 6-*O*-methyl-β-d-galactose (Table 1), 6-*O*-sulfo-α-l-galactose, and 3,6-AG units.^120^ Differences in the composition occur depending on the species. However, it was reported that, in nature, the sum of the β-d-galactose and 6-*O*-methyl-β-d-galactose is equal to the sum of the 6-*O*-sulfo-α-l-galactose and 3,6-AG units.^121^ Porphyran can only form hydrogels after an alkaline treatment that removes the sulfate groups on the polysaccharide backbone.^122,123^ While the modification of the backbone is necessary, the gelation is a physical process, and it does not need any additional reactive species, such as methacrylate groups used in synthetic and chemical hydrogels. This alkaline treatment is also often used during processing of agarose and carrageenan, converting the 6-*O*-sulfo-l-galactose into 3,6-AG. Thereby, the mechanical properties of the hydrogel are generally improved by “dekinking” the backbone and thus allowing longer helical structures to form.^124^

Once the sulfate groups are removed, porphyran gelation occurs through the aggregation of double helices.^123^ Only a few studies have been published on porphyran, and therefore further work is required to better understand its gelation mechanism and the factors influencing its hydrogel properties. This will be helpful to fully exploit its physical and biological properties for applications in biomaterials.^125^

### Colloidal Assembly

Within the family of hydrogel-forming polysaccharides extracted from algae presented and discussed herein, nanocellulose is the only polysaccharide having a colloidal-based gelation mechanism. Cellulose is composed of β-d-glucose units and can be obtained from various sources including plants, algae, and bacteria. Bacterial cellulose is a native strong, irreversibly entangled hydrogel,^126,127^ while algae and plant cellulose needs to be processed into nanocelluloses to form a hydrogel. Nanocelluloses are colloids, solid nanoparticles homogeneously dispersed in aqueous media. They are obtained through a deconstruction of the cellulose fiber into individual nanosized building blocks, which can be dependent on the treatment, either cellulose nanofibers (CNF) or nanocrystals (CNC).^128^ These colloids feature a fluid-like character in a diluted state and have a gel-like behavior at higher concentrations.^129^ The transition from the diluted state into a gel is reversible and based on repulsive particle–particle interactions.^13^ Hydrogels are formed upon a concentration threshold of the colloid, that is, critical concentration, which is mainly dependent on the aspect ratio and volume fraction of the colloid. In the case of CNF, the individual nanofibers form entanglements, and thus their aspect ratio and flexibility can favor the hydrogel formation.^130,131^ The colloidal characteristic of the hydrogel formed by nanocellulose confers their shear-thinning properties.^132^ Such flow properties make nanocelluloses easily processable as a gelled material and allows the embedment of living cells for injection into animals.^133^ This shear-thinning property is also an attractive attribute as rheology modifier in 3D printing inks.^134^ However, in contrast to other polysaccharide gels, such as agarose, native nanocellulose in the hydrogel state lacks a physical stability and is dispersed upon dilution. Thus, to overcome this limitation, nanocellulose is often combined with other hydrogel-forming polysaccharides extracted from algae.^135,136^

## Algae Culture and Extraction

According to Food and Agriculture Organization (FAO) statistics,^137^ farmed seaweeds provided 97% of the total annual world production of algae in 2018, with a weight of 32.4 million wet tons. Seaweed aquaculture is mostly located in the East and Southeast Asian countries of China, Indonesia, and Philippines. Although ∼220 species are cultivated worldwide, only six genera of seaweeds provide more than 95% of global farmed seaweeds production: Saccharina, Undaria, and Pyropia are essentially for food applications, and Eucheuma/Kappaphycus and Gracilaria are mainly used for carrageenan and agar extractions.

Farmed seaweeds are predominantly provided by ocean-based systems. At sea, depending on species, seaweed can be produced either on the seabed, attached to a hard substrate, or on flexible anchored lines or nets that are seeded. The economic viability of the offshore farming systems remains a challenge, since it faces many issues.^138^ For offshore farming seaweeds must be robust, resisting diseases and the growth of epiphytes throughout the seasons;^139^ the culture site can be exposed to extreme effects of weather and ocean conditions and is subjected to varying environmental conditions.^140^ For these reasons, offshore cultivation currently relies on a few robust seaweed strains, providing a large volume of monospecific biomasses at low cost while offering a weak diversity of algal raw material with varying quality, which is not necessarily suitable for the development of high-value biomaterials. Recently, the production of seaweed in natural reserves where the water quality is controlled and the shore preserved from industrial activity has emerged as a potential source of high-quality seaweed. This approach offers a valuable alternative to the costly land production while providing the necessary water quality.

Land-based seaweed cultivation takes place in closed systems such as tanks, raceways, ponds, or lagoons. In most cases, water is maintained under agitation to keep seaweeds freely suspended and exposed to the light. A broader diversity of seaweed genera (except the largest kelp species) can be produced this way with a higher yield per area compared to offshore systems. Onshore systems offer a high level of control over environmental conditions including nutrients, CO_2_, salinity, pH, and even light and UV exposure in some cases. Moreover, specific seaweed genera can be selected to obtain targeted biopolymers or chemical compounds.^140^ However, infrastructure building and the maintenance of farm conditions have a higher cost compared to offshore culture, and the availability of land and suitable water quality is limited. A major advantage of land-based algae farms, over harvesting, is the possibility of producing more standardized biomasses, whose chemical composition is more predictable, thus meeting the requirements to produce high-value seaweed ingredients for new markets.

## Industrial Production of Algal Polysaccharides

Each of the major algae polysaccharide families (agar, alginate, carrageenan) is produced industrially at a large scale since the first half of the twentieth century. While the processes vary for each polysaccharide type and also depend on the species used as a raw material, they are all based on several common features exploiting the ionic nature of the polysaccharide (pH adjustments, ion exchange, precipitation) and hydrogel properties (gelation), which can be preceded by an alkaline treatment to improve gelling properties and isolation.^141^ While these processes have been optimized over the years, they have seen few fundamental changes. This is mostly due to the limited amounts of new factories, since heavy investments are required to redesign an industrial process while the selling price of algal polysaccharides is generally low.

### Alginates Production

Alginates are extracted primarily from harvested brown seaweed species, although cultivated *Laminaria japonica* is sometimes used in China. The chopped seaweed is first lixiviated in acidic conditions to convert all the alginates in the seaweed into alginic acid and to extract undesirable compounds and minerals. A subsequent treatment in alkaline conditions allows its extraction as a viscous sodium alginate solution, which is diluted, optionally bleached, and filtered. The solution is then directly acidified to form alginic acid or undergoes an intermediate step of gelling as calcium alginate before conversion. The purified alginic acid can be used as it is or converted into sodium alginate or other types.^142^

### Agar Production

Most agar is produced from species of *Gelidium* and *Gracilaria*, using a similar process relying on hot water extraction but with different pretreatments.^142^*Gelidium* is directly heated in slightly acidic conditions, while *Gracilaria* is first treated in alkaline conditions to increase its 3,6-AG content and washed.^141^ Agar extraction is performed in hot water and followed by filtration to remove seaweed residues. The agar solution is subsequently cooled to form a gel (with subsequent washing and bleaching steps). Water is then partially removed by freeze–thawing or pressing of the gel, which is then dried and milled. In the course of this treatment, agaropectin can be, for example, removed through precipitation with poly(ethylene glycol) to obtain a pure agarose polysaccharide.^143^

### Refined Carrageenan Production

Carrageenans are mostly produced from cultivated *Kappaphycus* and *Eucheuma* species, but some harvested species such as *Chondrus crispus* or *Gigartina sp* are still used too. The production process for refined carrageenans is similar to the one used for agar. The washed seaweed is cooked in alkaline conditions to increase the 3,6-AG content and to extract the carrageenans. In a following alkaline treatment, the polysaccharide solution is filtered to remove seaweed residues and preconcentrated. Carrageenans can then be precipitated by an isopropyl alcohol addition and subsequently separated, pressed, washed, dried, and milled. Alternatively, κ-carrageenans can be gelled using potassium chloride and then processed as the agar gel.^142^

### Nanocellulose Production

Nanocellulose can be extracted from leftovers of the industrial extraction processes of algal polysaccharide. This has been demonstrated by, among others methods, the utilization of brown algae waste after alginate extraction for the isolation of high-aspect-ratio cellulose nanofibers.^144^ Hence, the integration of a cellulose production stream into existing industrial algae processes or valorization of solid waste streams is very feasible and already demonstrated for other algae species.^145^ To remove noncellulosic polysaccharides and other residues, a cellulose-rich fraction from algae is purified by (1) an extraction of lipids, (2) NaOH treatment, and (3) bleaching steps.^144,146,147^ Hydrochloric acid treatments are optional and can be added to increase further the cellulose purity.^144,148^ These purified cellulose fractions can be then processed into hydrogel-forming CNF or CNC. CNF were obtained from algae by a mechanical high-pressure homogenization^147^ or by a (2,2,6,6-tetramethylpiperidin-1-yl)oxidanyl (TEMPO) oxidation and subsequent ultrasonication treatment.^11,144,149^ These processes cause a fibrillation of the algal cellulose fiber into individual nanofibers. CNC were obtained by an acidic hydrolysis of amorphous, disordered regions of cellulose, by sulfuric acid treatment.^150^ Algal nanocellulose possesses a significantly higher aspect ratio than woody nanocellulose and shows, hence, a high potential for the production of mechanically robust hydrogels. Algal CNFs have already been explored as a scaffold for human dermal fibroblast cells and have been shown to promote fibroblast adhesion and support high cell viability.^11^ Purification processes have been as well established to produce high-purity algal nanocelluloses for biomedical applications.^148^

## Emerging Algal Polysaccharides Extraction Methods

**Figure 2:**
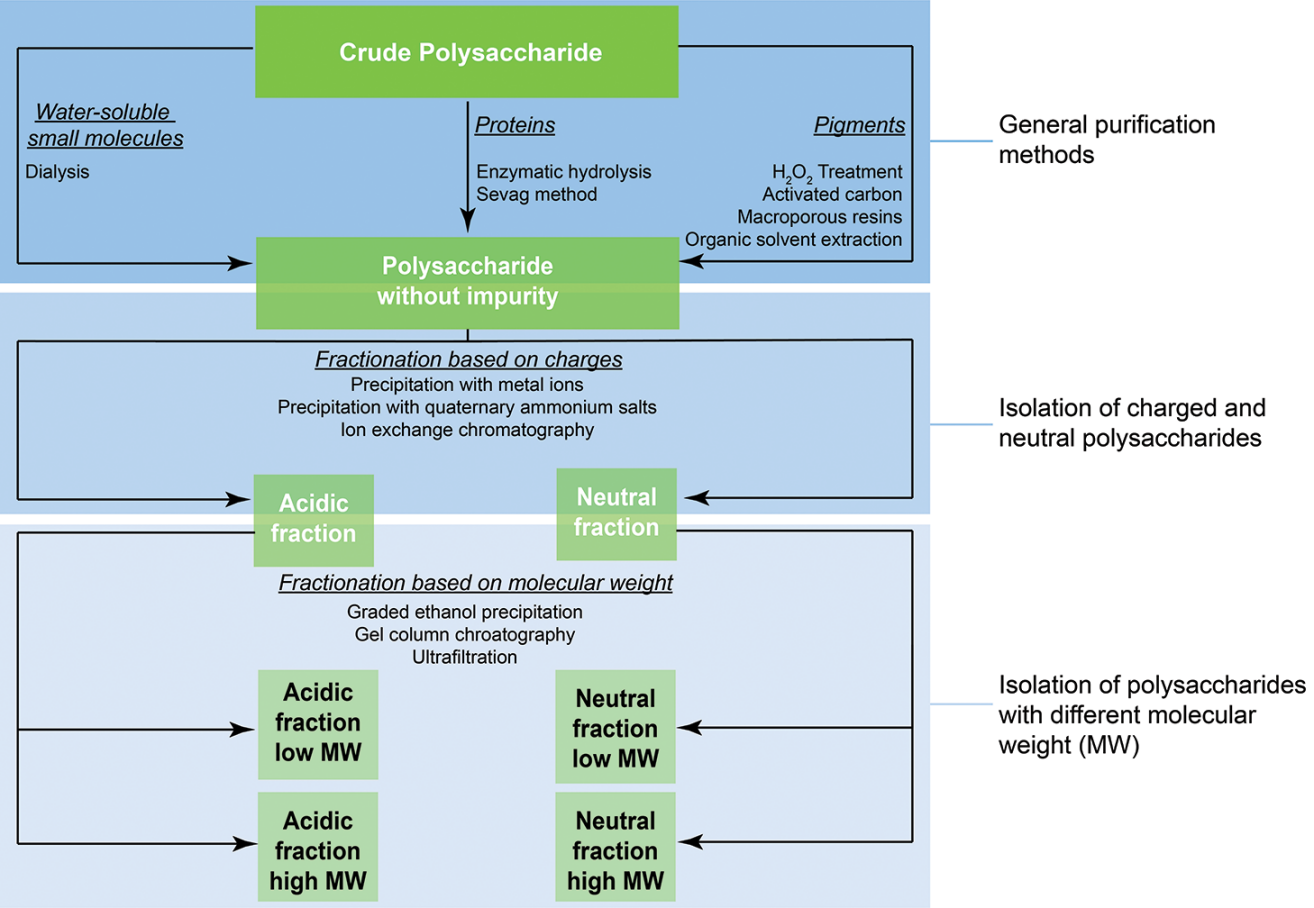
Purification and extraction routes that can be used to isolate a polysaccharide according to its chemical structure.

As the need for algal polysaccharide is growing, processes to extract novel polysaccharides are emerging. These may include neutral algal polysaccharides as, for example, laminarin, or complex ionic fucoidan and fucose-containing seaweed polysaccharides (FCSPs) from brown species, or ulvans, whose composition will strongly depend on the species used and thus require modification of the known processes to adapt to the specificities of the algae species. The use of acidic or alkaline conditions, commonly performed for a polysaccharide extraction, might also impact the molecular weight or sulfation degree, hence impacting the hydrogel properties or biological activity.^151^ A schematic overview of extraction strategies for algal polysaccharides is given in Figure 2. However, new advanced extraction techniques (some not yet fully available at industrial scale) are also increasingly explored to improve yields or selectivity of the extractions. Obtaining a pure material often requires additional steps to remove small impurities (heavy metal, low molecular weight polymer, pigments). Dialysis can easily remove small water-soluble impurities such as salts by using a membrane with a suitable molecular weight cut off (MWCO). At a lab scale, the removal of protein is usually achieved by following the Sevag method, which uses a mixture of chloroform and *n*-butanol to denature and thus separate proteins from aqueous polysaccharide solutions.^152^ Because of the toxicity and environmental impact of chloroform, its use is strictly regulated. Alternatively, enzymatic hydrolysis is often used, which is highly efficient under mild conditions.^153^ In many cases a combination of both methods can lead to an increased effectiveness of protein removal.^154^ With the development of biotechnology, more advanced and pure enzyme cocktails can be obtained for the selective removal of polymeric residues. One example is the enzyme-assisted extractions using a commercial “terrestrial” enzyme, such as cellulases, that can be leveraged to selectively isolate a hydrogel-forming polysaccharide.^155−157^

Phenolic substances in the algae can cause an undesirable coloring of the polysaccharide extract. Obtaining a colorless material can be achieved by bleaching with hydrogen peroxide,^158^ extraction with organic solvents,^159^ or purification using macroporous resin.^160^ Note that too-high concentrations of hydrogen peroxide can lead to a decomposition of the polysaccharides reducing its molecular weight and the extraction yield.^161^ The extraction of polysaccharides can rely on the chemical properties, such as charge, for alginate.^162^ More recent advances based on flocculation processes are using long-chain quaternary ammonium salts, to precipitate the polysaccharide by the formation of water-insoluble complexes and separate the complex from neutral biopolymers. For this method, commonly used reagents are hexadecyltrimethyllammonium bromide^163,164^ and cetylpyridinium chloride.^165^ Besides precipitation by long-chain quaternary ammonium ions, ion-exchange chromatography is also being developed for polysaccharide purification. While this purification method can be time-consuming, the obtained products are of high purity,^166,167^ which can be particularly attractive for high-value polysaccharides for food or biomedical applications. In addition to the industrial ethanol fractioning methods,^168,169^ other important purification methods are based on ultrafiltration^170^, size-exclusion and affinity chromatography^171^ as well as solid-phase extraction.^157,172^

## Characterization of Hydrogel-Forming Polysaccharide

Algae polysaccharides are extracted from the complex algae matrix by a deconstruction into its polymeric components, which is a top-down approach. This stays in contrast to synthetic polymer obtained by a bottom-up synthesis: from monomers to polymers. Because of the complexity of biomatrices, such as algae, the extraction process is very important, and it is necessary to determine the exact composition of the extracted biopolymer, which might contain impurities in the form of proteins, lipids, polyphenols, or inorganics (heavy metals).

**Figure 3:**
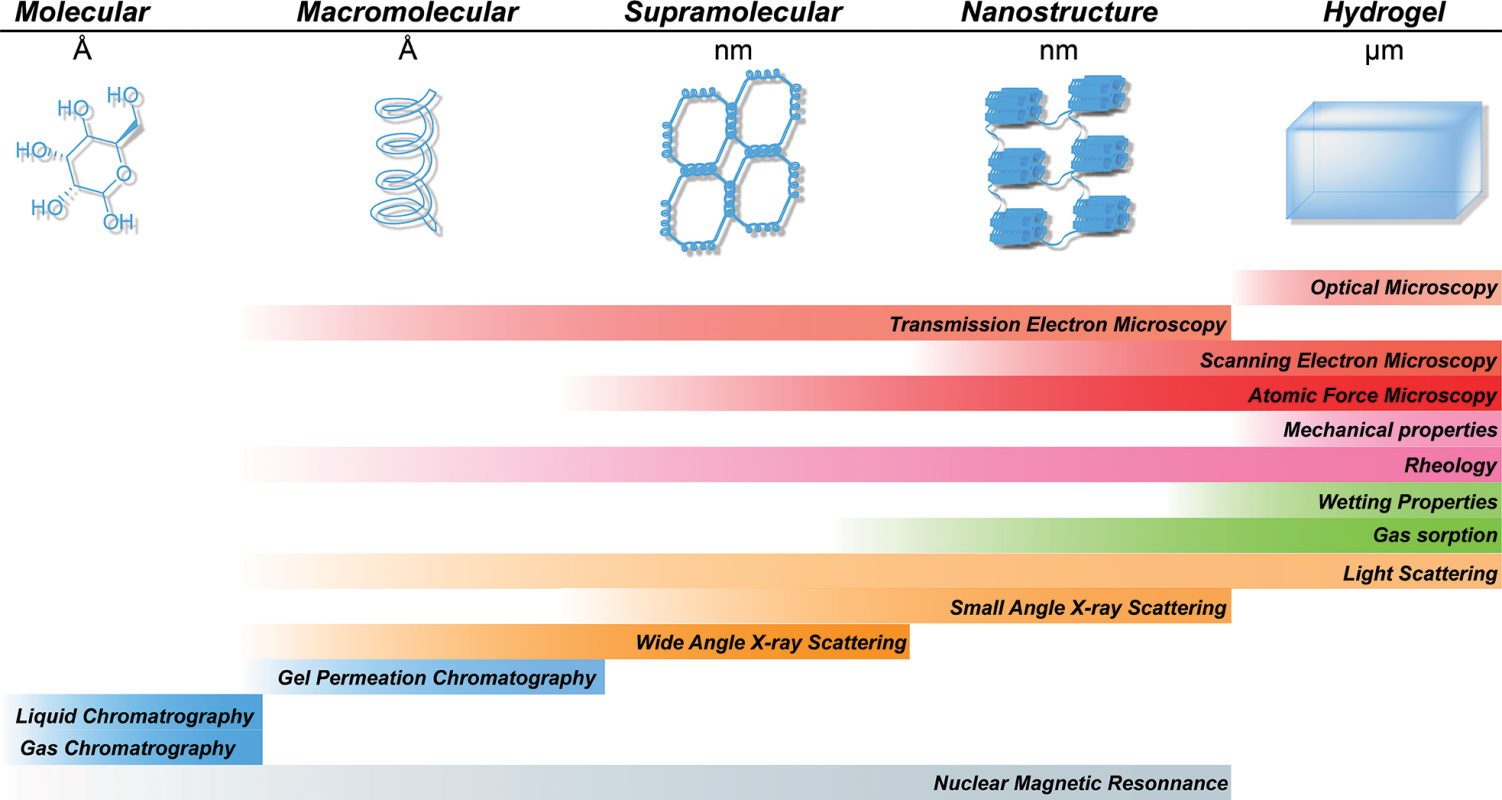
Analytical techniques available for the characterization of hydrogel-forming polysaccharides from algae. Techniques are classified by the scale of the characterized structure. Gray: nuclear magnetic resonance spectroscopy, blue: chromatography, orange: scattering techniques, green: gas and liquid sorption, pink: mechanical properties, red: microscopic techniques.

Similarly to the case of synthetic polymers, several characterization techniques are available to determine the chemical composition and structure of algae-extracted polysaccharides. The thorough characterization of the chemical structure, up to the hydrogel physical state, should enable a linking of the chemical properties to the performance of the resulting hydrogel (Figure 3), and this is critical to define the potential use and applications of these polysaccharides.

### Molecular Scale

The monomer composition of the polysaccharides dictates its final properties. However, an analysis of the monomer units can be challenging when polymers are branched or in the case of complex polysaccharides such as carrageenans. The sugar composition is usually studied by the analysis of monomeric sugars obtained from a two-step acidic hydrolysis, i.e., total hydrolysis, in which the carbohydrates are first prehydrolyzed in a 72 wt % aqueous solution of H_2_SO_4_ and then further hydrolyzed into the individual repeating units in more diluted H_2_SO_4_ at 40 wt %.^173^ Then the monomeric sugars are analyzed by ion chromatography coupled with a refractive index detector,^174^ gas chromatography (GC) coupled with mass spectrometry,^175^ or a flame ionization detector.^176^ In the case of GC analysis a prior derivatization step is required to increase the volatility of the analytes, in most cases by a silanization of the saccharide.^177^ Following these techniques the amount of each sugar of the polysaccharides can be assessed, but one must take into account that less common sugars or generally charged sugar units might not be detectable by a standard method and will require more specifically optimized techniques. After a sugar hydrolysis, monomeric sugars can also be studied by liquid-state nuclear magnetic resonance (NMR) spectroscopy to determine their structures as well as identify the number and type of functional groups.^178,179^ Further structural information can be obtained by including desulfation^181^ and methylation^182^ steps prior to the analysis.^183^ NMR analysis can be also performed on oligomeric fractions, obtained via enzymatic hydrolysis.^184^

More conventionally, polysaccharides can be analyzed by colorimetric methods. These methods are simple, as they do not need special equipment and are often used in the case of agarose and polysaccharides for food applications.^185,186^ In these examples, the total carbohydrate content is determined by the phenol-sulfuric acid method of Dubois et al.;^187^ in which polysaccharides are hydrolyzed into their repeating units and further reacted with phenols to form conjugated molecules with a yellow-gold color. The total carbohydrate content can then be quantified by measuring their absorption in the visible light range and relating this to suitable calibration curves. The amounts of 3,6-AG units can be also quantified by a colorimetric method, known as the resorcinol method. This method is based on the reaction of resorcinol with ketose via the Seliwanoff reaction and works with ketose sugars formed in the dehydration of 3,6-AG.^188,189^ To measure the sulfate contents, the simplest methods are based on turbidity measurements using BaCl_2_ causing the precipitation of the insoluble BaSO_4_, which is formed from the sulfate ions in polysaccharide hydrolysates.^190,191^ In this method gelatin is often added to stabilize the BaSO_4_ suspension and improve thereby the reliability of the measurement. Generally, for polysaccharide containing charged repeating units, these units can be quantified by a conductometric titration.^192^ Since sulfate and carboxylate have different p*K*_a_ values, they can be determined simultaneously by this method,^193^ which is especially interesting for complex polysaccharides or chemically modified ones. In addition to the monomer composition, a trace element such as a heavy metal can be detected by an elemental analysis and help to determine the purity of the polysaccharide extract.^194^

### Macromolecular Scale

At the macromolecular scale, the length of the polymer chains is the most critical characteristic that can dictate many properties of the polysaccharide. The polymer length can be expressed as the degree of polymerization (DP), that is, the average number of monomer units per polymer chain, but it is commonly described by the number-averaged (*M*_n_) and weight-averaged molecular weight (*M*_w_). The latter can be directly determined via light scattering.^195^ The *M*_w_ of commercially extracted polysaccharides can greatly vary due to occurring polymer degradation during extraction and purification processes.^196,197^ In general, the higher the purity, the lower the final DP of the purified polysaccharide. This is because of the recalcitrant algae matrix, which requires harsh extraction and purification conditions, which can cause polymer degradation via chain scission.

While various techniques are available to determine the absolute molecular weight of polysaccharides, the preferred methods are based on light scattering.^195^ A combination of light-scattering techniques with a previous separation method based on size, such as gel permeation chromatography (GPC) or size exclusion chromatography, gives not only averaged molecular weight numbers but also the molecular weight distribution and polydispersity values. Alternatively to get the absolute molecular weight measurement, GPC equipped with a refractive index detector can be used to determine the relative molecular weight of the polysaccharide by applying a calibration curve of polymer standards with uniform molecular weights, typically pullulan or dextran.^26^

### Supramolecular Characterization

The interactions of individual biopolymer chains can result in supramolecular assemblies. These structures can be classified as having a short-range order, and one distinguishes it mostly between an α-helix and β-sheet.^198^ The secondary structure of biological molecules is commonly studied by circular dichroism (CD).^199^ For instance, an agarose secondary structure can be characterized by CD. The signal arises from the coupling of C–O–C ether chromophores, leading to a positive residual ellipticity.^200^ Alternative techniques to study the secondary structure of polysaccharides and its effect on their properties are based on optical rotation.^112,201^

In polysaccharides with crystalline domains, mostly cellulose, the crystallinity index and crystal dimensions are conventionally measured with wide-angle X-ray scattering (WAXS) and are based on the scattering of the X-rays in diffraction patterns. The intensity of these patterns relates to the overall crystallinity index, which can be determined by a subtraction of the broad amorphous peaks, and the crystallite size can be determined from the peak width.^202^ In addition, the chemical and physical environment of polysaccharide chains in crystalline domains is different from the one in amorphous regions; this affects the chemical shift of characteristic peaks in solid-state NMR^203−205^ as well as the wavenumber of the bands from IR^206,207^ and Raman^208^ spectra, and can be used to estimate the sample crystallinity. Nanoparticle dimensions and aspect ratios are mostly determined by using atomic force microscopy (AFM) and transmission electron microscopy (TEM). In all these methods, the sample preparation plays a crucial role, and it is recommended to follow well-established protocols, to make sure that the analyzed nanoparticle fraction represents the whole sample; alternatively, also scanning electron microscopy (SEM) can be used to measure the dimensions of polysaccharides organized into nanoparticles.^194,209^ The size of nanoparticle or polysaccharide aggregates can be as well determined by dynamic light scattering (DLS) measuring the time-dependent fluctuations of the scattered light intensity of particles.^210^ This is based on a simplification to a spherical shape, hence the obtained size is in the case of differently shaped particles not absolute, but it can be used as to assess the state of dispersion and the hydrodynamic radius of the nanoparticles.^211^ Apart from crystallinity and dimensions, the colloidal stability of polysaccharide nanoparticle electrostatic polymer chain repulsion or particles can be assessed in the form of the zeta potential^194,212^ usually measured by electrophoretic light scattering. This is based on the determination of the electrophoretic mobility of a nanoparticle in an applied electric field determined by light scattering, which can be then converted to the nanoparticle’s zeta potential using the Henry equation with Smoluchowski or Huckel approximations.^213^

### Nanostructure Characterization (Dry Hydrogel)

As introduced in the previous section, SEM and TEM can be used to investigate the nanostructure of supramolecular aggregates.^194,209^ But these electron microscopy techniques can also be used for the structural analysis of dried hydrogels. In this case the type of drying procedure is of utmost importance. The goal of these drying procedures is to produce a sample, from which water is replaced by air without causing structural changes to the sample, and the obtained highly porous dried hydrogel is referred to as an aerogel.^128^ The most-used drying technique for hydrogels is freeze-drying. It is a well-established method used, for example, in food and biological applications and can also be used in an industrial scale.^214,215^ In this process the hydrogel is frozen, and the frozen water is removed under a high vacuum by sublimation.^216^ However, an agglomeration in the hydrogel occurs during this method in the freezing step.^217,218^ In the case of water as the liquid phase, the formed ice crystals push the hydrogel nanostructure together and induce thereby agglomeration into a sheetlike structure, which is not very representative of its solution nor native solid state.^217,219,220^ The size of the ice crystals is dependent on the freezing procedure and also on the sample thickness, and can be reduced by solvent exchange to *tert*-butanol.^14,221^ Freeze-drying from *tert*-butanol yields structures that are very similar to aerogels obtained from a supercritical CO_2_ drying (or a critical point drying).^128,221^ In the case of a supercritical CO_2_ drying, a prior solvent exchange to EtOH or acetone is usually conducted, and the drying yields a representative aerogel.^216,222,223^

Once appropriately dried, the nanostructure of the polysaccharide or hydrogel can be investigated by high-resolution electronic microscopy. However, polysaccharide aerogels are nonconductive insulators. If not properly handled surface charging can lead to a loss of contrast and difficulties in the acquiring of images. Moreover, the high voltage of the electron beam can cause local damage and a structural alteration of the delicate aerogel structure. To overcome these issues, one can coat the specimen with a conductive layer, conventionally gold, platinum, or iridium.^194,209^

While electron microscopy can provide information on the structure of the polysaccharide the size of the pore and formed structure can be challenging to measure. The specific surface area can be determined by gas sorption measurements of the aerogels with nitrogen as the most common sorbate. The surface area and pore size distribution are in this case calculated from nitrogen sorption isotherms according to Brunauer, Emmett, and Teller (BET) and Barrett, Joyner, and Halenda (BJH) theories, respectively.^224,225^ These techniques have been extended to the sorption of different gases such as octane, which can be performed at room temperature.^226^ Limited to a nanosized pore size (<100 nm), gas sorption cannot measure micrometer-scale pores. Mercury intrusion is better-suited to analyze a broad pore size range of up to the size of several hundred microns.^227^ Alternative noninvasive methods such as microcomputed tomography can analyze pores in micrometer and centimeter ranges and provide an image from which tortuosity and pore interconnectivity can be calculated.^228,229^

### Hydrogel Characterization

While the previous characterization techniques focused on the characterization of the hydrogel in the dry state, these can be destructive. For some applications, noninvasive, nondestructive techniques are needed for the characterization of the polysaccharides in the hydrated hydrogel state. A direct analysis of the hydrogel nanostructure by AFM has been shown for polysaccharide and protein hydrogels.^230^ AFM force measurements can also reveal information on the mechanical properties of the nanostructures.^231^ In comparison to SEM, no special sample preparation is necessary, but the drawbacks of AFM are the time-consuming measurement and the relatively small measured sample areas.

Small-angle X-ray scattering (SAXS) can reveal information on the gel properties, such as the alignment, fibrillar diameter, or specific surface area.^14,232^ The measurement principle is similar to that of wide-angle X-ray scattering (WAXS), but it is sensitive to larger aggregates, such as nanofibers. A SAXS diffraction pattern is dependent on the scattering on these structural motifs, and a fitting and analysis of these patterns gives detailed structural information. In the wet state, the pore size of a hydrogel structure can be imaged by magnetic resonance imaging, which is a noninvasive and nondestructive method that does not use ionizing radiation.^233^ Alternatively, X-ray tomography can be used in wet conditions but requires the application of contrast agents.^234^ Another method to determine the pore sizes of hydrogels is to determine the hydrogel permeability to defined polymers with a known hydrodynamic radius.^235^

The water content of hydrogels can vary depending on the hydrogel environment. Therefore, it is important to measure their swelling behavior, usually expressed by the swelling degree, that is, the amount of water per unit mass of the dry sample.^236^ The change of water content in a hydrogel affects their mechanical properties, and hence the mechanical properties of a specimen should be always measured in a swollen and equilibrated state to provide a representative value of their performance during the attended application.^194^ The simplest measurement to compare the mechanical properties of hydrogel is unconfined compression testing, using a standard universal tensile testing machine equipped with compression plates, a texture analyzer, or a similar setup. Thereby, the mechanical properties of a whole hydrogel sample can be determined, and the compressive elastic modulus can be extracted from the initial slope of the compression test curve. This modulus gives information about the viscoelasticity of the given sample; in addition, cyclic compressive tests can be conducted to assess the elasticity of the hydrogel and its long-term stability under stress. Depending on the sample and application, an even smaller-scale mechanical analysis, such as nanoindentation with AFM, can be useful to reflect the mechanical resistance on a supramolecular level.^237^ Determining the stiffness at this scale can be of interest to reflect the sensing behavior of living tissue, as living cells can feel and respond to the stiffness of a substrate material.^238^ The viscoelastic properties including the storage and loss modulus of hydrogels can be further characterized by dynamic measurements, such as a dynamic mechanical or rheological analysis. The storage modulus describes the elastic behavior, and the loss modulus describes the viscous behavior of a sample. These moduli are used to give the definition of a gel. A gel is classified as a soft solid with a higher elastic comportment than viscous behavior; analysis of these moduli gives important information on the gel strength and network interactions.^239^

## Chemical Modification

The chemical structure of algae-extracted polysaccharide governs their abilities to form hydrogels. Understanding the relationship between their chemical structure and physical properties enables the prediction of certain polysaccharide properties upon chemical modification. Nevertheless, the available functionality of these natural polymers and the reactivity are rather limited (Table 2). A chemical modification of the accessible repeating units by a controlled introduction of different functional groups allows us to tune the hydrogel formation, to perform coupling chemistry; and to bind biological molecules such as peptides^198^ or reactive groups such as acrylates for the chemical cross-linking of the polysaccharide.^240^

**Table 2:**
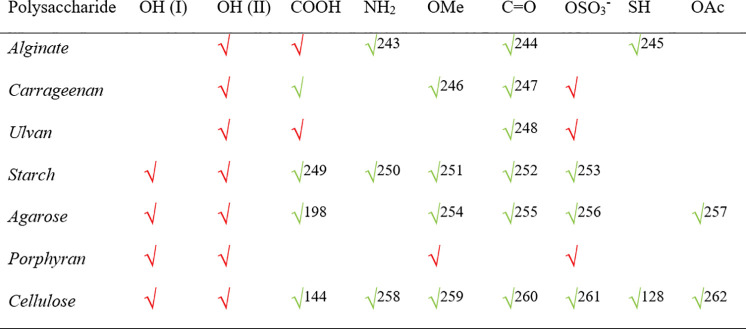
Functional Groups Naturally Found on Algal Polysaccharides (red √) and Reported^a^Chemical Backbone Modification (green √)

a Numbers in the table indicate reference citations.

Naturally, these polysaccharides all bear secondary hydroxyl groups. Some, such as starch, agarose, porphyran, and cellulose contain primary ones. These primary groups are generally more reactive than secondary alcohols^179^ and can be oxidized regioselectively to carboxylic acids.^241^ In comparison to the hydroxyl group, the natively available carboxyl in alginate and ulvan is more reactive and can be directly used for a peptide coupling via amidation.^242^ Moreover, the amount of functional groups, such as sulfate and methyl groups in porphyran, has been shown to influence its gelation properties. This knowledge relates the chemical structure with the physical properties of natural polysaccharides and can be used to fine-tune the physical properties of these hydrogels via chemical backbone modification.

**Figure 4:**
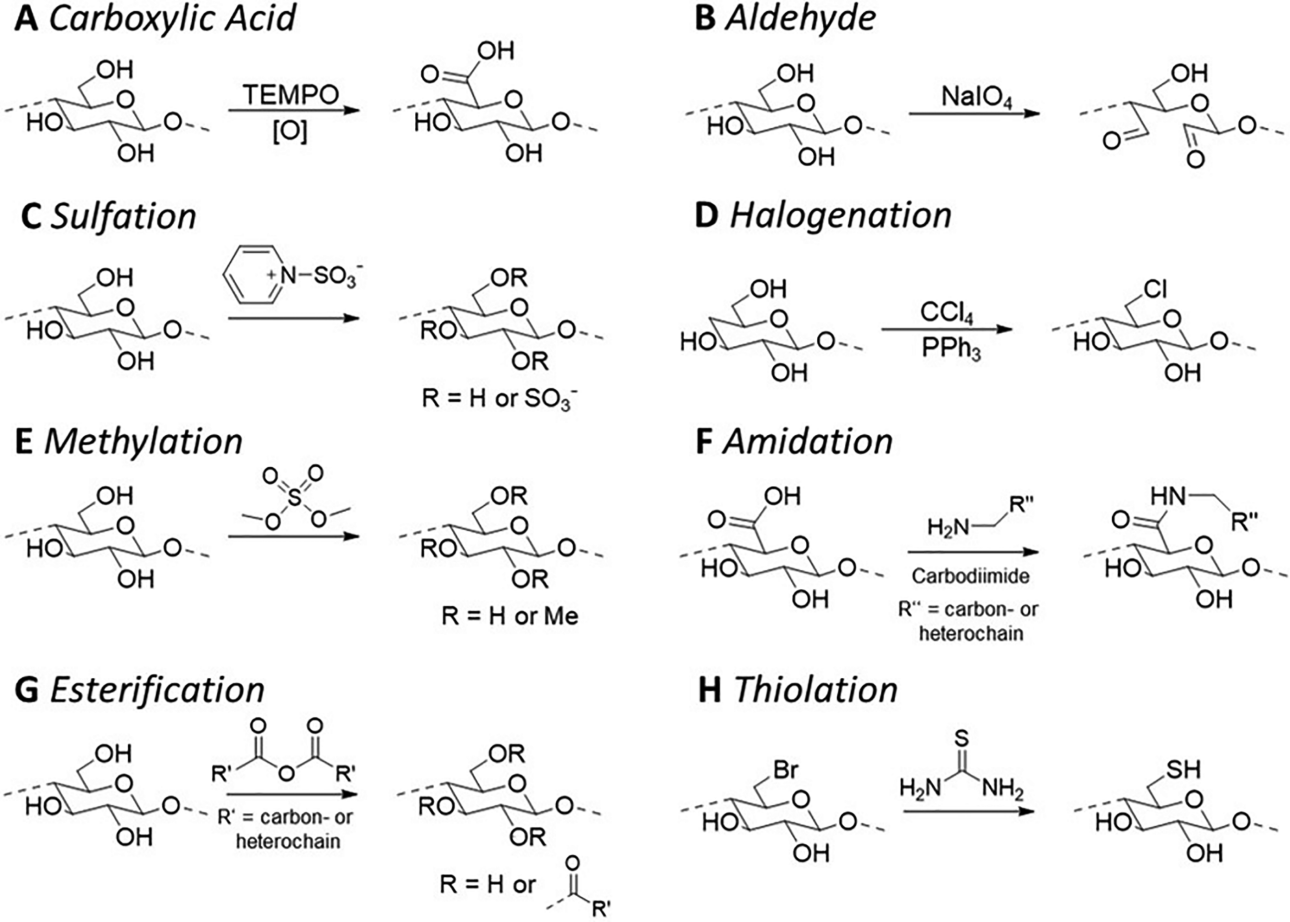
Reported chemical reactions to modify an algae polysaccharide backbone. The reactions are shown exemplified on glucose or glucuronic acid building blocks.

The natively occurring chemical groups of selected algal polysaccharides and the most frequently introduced functional groups are summarized in Table 2, and we present the basic chemical reactions to introduce these functional groups in Figure 3.

(A) ***Carboxylic acid*** functional groups are attractive for the functionalization of polysaccharide hydrogels. Indeed, they enable the coupling of biological signals, such as short peptides as demonstrated with alginate.^263^ In contrast, polysaccharides without these groups, e.g., agarose or cellulose, are challenging to functionalize. An attractive and straightforward avenue is the oxidation of their primary alcohol into a carboxylic acid. The most studied reaction for the mild oxidation of a polysaccharide is the TEMPO-mediated oxidation (Figure 4A). TEMPO-mediated oxidation is mostly conducted in the presence of NaBr and the oxidizer, chlorine, under alkaline conditions.^241,264^ But it can be also conducted in neutral conditions.^265^ It is important to take into account that the introduction of a carboxylic acid onto the backbone of agarose and other polysaccharides can modify their secondary structure and thereby their gelation mechanism;^198^ in addition, the molar mass of the polysaccharide is reduced due to the accompanying chain degradation.^266^ A TEMPO-mediated oxidation plays also an important role in the preparation of CNF as negatively charged carboxylate groups are introduced, which facilitate the deconstruction of the cellulose fiber into individual nanofibers.^144,267^
(B) ***Aldehydes*** offer also a highly reactive site for the functionalization of polysaccharides.^252^ These groups can be introduced in a straightforward manner via periodate oxidation. This oxidation is applicable only on adjacent hydroxyl groups; in the case of sugars it mostly attacks C2- and C3-OHs leading to the cleavage of the C2–C3 carbon bond and the formation of two aldehyde groups at C2 and C3 (Figure 4A).^268^ Consequently, it does not react with agarose but is frequently used for 1,4-linked glucose-containing polysaccharides, such as cellulose^260^ or starch.^269^ Recently, it was shown that the resource efficiency of the periodate oxidation can be tremendously increased by reaction at high solid content.^207^ In the case of cellulose, these oxidized groups can be postmodified to give access to CNF decorated with various functional groups, including, among others, carboxylate and sulfonate ones.^270,271^
(C) ***Sulfated*** polysaccharides can be made using sulfur trioxide pyridine, yielding a polysaccharide substituted with sulfate groups (Figure 4C).^272,273^ The addition of sulfate groups in a polysaccharide can help to mimic a naturally occurring backbone modification (e.g., in λ-carrageenan) that impacts the hydrogel formation. These sulfate groups add negative charges on the polysaccharide, which then can be used as a polyanion with antifouling applications.^274^ So these sulfated carbohydrate can provide anticoagulation properties, as it was reported for agarose.^256^ Cellulose nanocrystals prepared by sulfuric acid treatment are as well slightly sulfated.^275^ Alternatively, more hydrolytically stable polysaccharide sulfonates (carbon-linked SO_3_^–^) can be obtained, for example, via a periodate oxidation of cellulose followed by a reaction with bisulfites.^271^
(D) ***Halogenation*** of a carbohydrate can be achieved with triphenylphosphine and tetrachloride to introduce chloride groups (Figure 4D).^276^ Other protocols report the use of triphenylphosphine in the presence of imidazole and iodine to introduce iodide groups.^277^ The halogenation of polysaccharide is also achieved indirectly via esterification and is useful to introduce bromide groups for a grafting polymerization through a surface-initiated atom transfer radical polymerization.^278^ In addition to coupling applications, a halogenated polysaccharide could be useful for the creation of biocompatible polymers, which can form halogen bond interactions that are stronger than hydrogen bonds. Such bonding is expected to have, for example, applications in medicinal chemistry to create new inhibitor-based drugs.^279^
(E) ***Methylation*** of a polysaccharide can be performed using dimethyl sulfate under alkaline conditions (Figure 4E).^254^ This method was used on agarose to control its gelation properties; a higher methylation led to a lower gelling temperature and lower gel strength.^254^ The agarose methylation reproduces the natural gel strength regulation of agarose in the algae. Depending on the season and the area of the culture, agarose with a different methylation can be extracted. While the methylation of a polysaccharide such as agarose is an efficient strategy to control the hydrogel properties, it limits the reactivity of the resulting polysaccharide by blocking, among others, the C6-OH position of the monomer repeating units.
(F) ***Amidation*** of a carboxylic acid-containing polysaccharide can be achieved through carbodiimide chemistry (Figure 4F).^280^ Typically polysaccharides that can undergo amidation are alginate and ulvan through their C6 carboxylic acid group. Other polysaccharides bearing a primary alcohol on their C6 position of the repeating unit require a prior TEMPO oxidation or periodate oxidation of C2- and C3-OHs and a subsequent chloride oxidation prior to a reaction with amines via amidation. Amidation reactions are often used for peptide coupling but can be also used to introduce positively charged functional groups on the polymer backbone.^281^
(G) ***Esterification*** is one of the most conducted treatments of polysaccharides. The most common reaction is the acetylation of cellulose to cellulose acetate by an acid-catalyzed reaction with acetic anhydride (Figure 4G).^282^ The introduction of acetyl groups onto a polysaccharide increases the hydrophobicity of the polysaccharide and thus improves its processability in an organic solvent.^283^ Naturally, polysaccharides such as alginate or agarose do not promote cell adhesion. Thus, the acetylation of these polysaccharides is of interest to increase the hydrophobicity of such polysaccharides, which can induce the absorption of proteins on the polysaccharide backbone and subsequently cell adhesion. Recently, also a wet esterification process for cellulose has been developed^205^ yielding a surface-acetylated CNF.^262^ It is expected that this protocol will be also applicable to other polysaccharides.
(H) ***Thiolation*** of a polysaccharide can be achieved by using a reaction of thiourea with halogenated polysaccharides (Figure 4H).^284^ The presence of thiols group on the polysaccharide backbone has two major applications: One is for the reversible immobilization of enzymes through disulfide bounds,^285^ and the second is as a mucoadhesive polymer.^286^ With the increased demand of a mucoadhesive drug delivery system, thiolated cellulose systems have been shown to exhibit important adhesive properties while being able to encapsulate and release pharmaceutics. As the demand grows, additional thiolate polysaccharides are needed to uncover new applications and broaden the formulation potential. As such, alginate demonstrated also mucoadhesive properties once thiolated.^245^

On the basis of the presented chemical avenues, especially amidation, esterification, or aldehyde modification, it is possible to further functionalize the polysaccharide by introducing chemical anchor groups for postmodification. For example, with the introduction of azide groups, a desired functionality with an alkyne group can be introduced via a copper-catalyzed or strain-promoted azide–alkyne cycloaddition,^287^ both of which are highly efficient and versatile reactions classified as click chemistry.^288^ A click reaction can be also performed with thiolated polysaccharides via thiol–ene click chemistry, with strained double bonds, for example, norbornenes, by inverse electron-demand Diels–Alder reactions or with maleic anhydrides.^289^ These reactions are in many cases also bio-orthogonal and enable selective reactions in the presence of living tissue,^288^ and they can be used for rapid in situ functionalization and cross-linking of polysaccharides. Recently, an aqueous silanization protocol was established to introduce multiple functional groups onto (nano)cellulose in aqueous media using catalytic amounts of HCl and NaOH.^290,291^ This approach is a highly versatile method to introduce functional alkoxysilanes with azido, thiol, and other groups and lead to polysaccharides that can be postfunctionalized via click chemistry.^290,291^

## Processing of Algae Hydrogel-Forming Algae Polysaccharides

**Figure 5:**
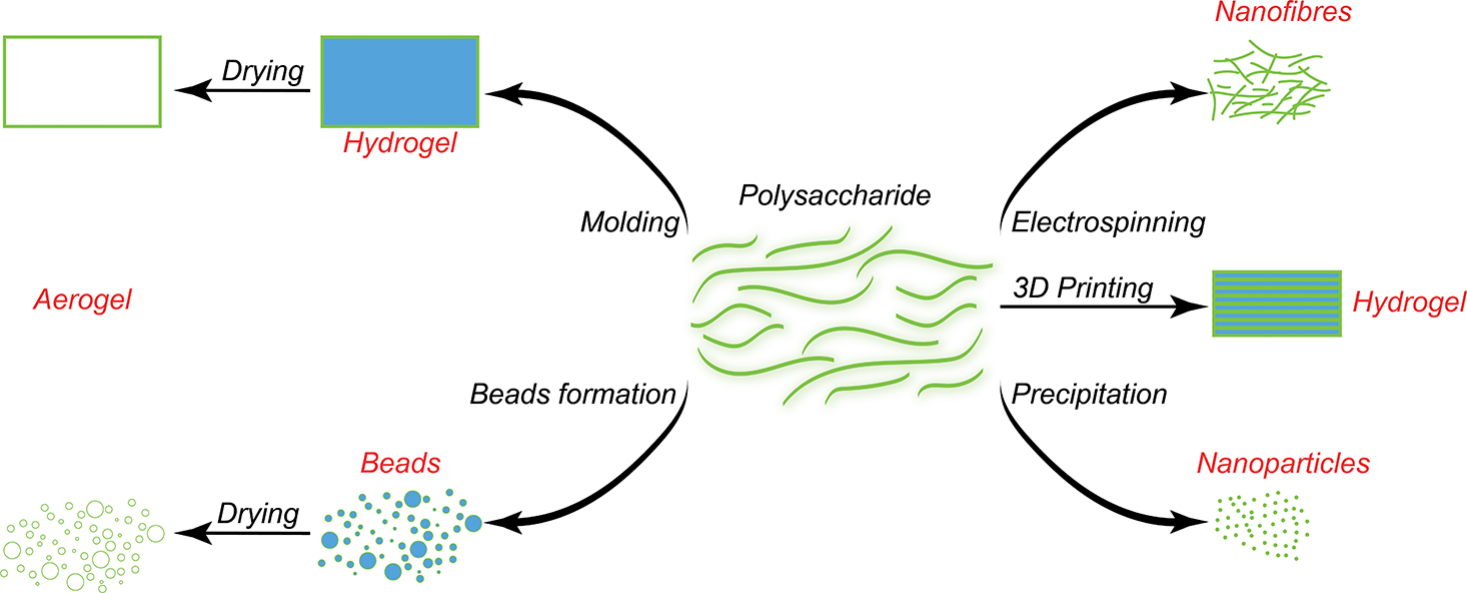
Common processing techniques of hydrogel-forming polysaccharides to create microbeads, nanoparticles, nanofibers, hydrogels, and aerogels that can be then used for biomedical applications.

Once the desired physiochemical properties of the algae-extracted polysaccharide are obtained, one must process the material into a shape, or morphology adequate for the targeted application. We identified the six most applied processing techniques for these hydrogel-forming polysaccharides: (a) molding, (b) 3D printing, (c) bead formation, (d) drying, (e) electrospinning, and (f) nanoparticle precipitation (Figure 5).

A ***Molding*** is the most straightforward processing technique to produce hydrogels into a desired form. Usually, a polysaccharide solution is injected or poured into a mold. Subsequently, the physical gelation is induced to form the hydrogel and retain its shape. The precision and structure of the final hydrogel is strongly dependent on the used polymer but enables even replication of microscale structures.^292^ For instance, agarose is used in soft lithography to obtain micrometer precision objects.^293^ Additionally, a molded hydrogel can be supplemented with biologically active molecules such as antibiotics to manufacture wound dressings.^294^ Sophisticated structures showing inner porosity^295^ or complex 3D structures can be obtained by sacrificial templating using sugar or salts, which can be removed without impacting the hydrogel stability or structure.^229,296,297^
B ***3D printing****or additive manufacturing* is a very useful method to prepare hydrogels with a specific shape without the use of a mold.^298^ This technique can be used to print a cell suspension in the hydrogel to prepare cell-laden hydrogels for tissue engineering. However, additive manufacturing techniques require a deep understanding of the polysaccharide rheological properties to adapt the printer flow to the solution viscosity. Some of the hydrogel-forming polysaccharide presented herein exhibit a shear-thinning property, that is, a reversible reduction of viscosity upon shear stress, and an intrinsic feature of many algal polysaccharides, such as agarose,^299^ alginate,^300^ and nanocellulose.^133^ This property makes them particularly suitable for applications requiring extrusion such as additive manufacturing. The shear-thinning properties of hydrogels can be further enhanced by an addition of rheology modifiers, such as silicates^301^ or nanocellulose,^302^ chemical modification of the polysaccharide,^303^ or a combination of algal polysaccharides.^304^ For instance, agarose is frequently used in combination with alginate for bioprinting without requiring any additional ionic cross-linking.^304^ Alginate by itself has a limited mechanical stability, and usually 3D-printed objects require post gelation with Ca^2+^, which reduces the practical suitability of this polysaccharide for bioprinting applications. But combined with nanocelluloses the shape fidelity and structural integrity of printed hydrogels is extremely improved.^136,302,305^
C ***Bead formation*** is particularly useful for drug delivery applications. These microbeads can be manufactured by simply dropping the dissolved polysaccharide into a solution that triggers the gelation process while maintaining the drop shape. The shape of these beads is controlled by the applied pressure during the extrusion, type of nozzle, and droplet size. κ-Carrageenan beads with a size of 22–32 μm were produced through an extrusion from a needle (0.6 mm diameter) into a solution containing potassium ions inducing gelation.^306^ Analogously, alginate beads can be prepared by physically cross-linking in CaCl_2_ solution, and drugs can be incorporated directly into the beads.^307^ The main challenge of this bead production technique is the control over the bead size and shape. But this issue can be overcome by using microfluidic techniques, which enable the preparation of highly uniform spherical particles using a polysaccharide solution and a nonmiscible oil phase in the presence of surfactant.^308−311^
D ***Drying*** of hydrogels is usually conducted via freeze-drying to produce highly porous structures, that is, cryogels. Dry gels can be more easily stored and sterilized than their hydrogel counterparts. But freeze-drying the hydrogel generally modifies its structure due to ice formation. This can be used to induce a controlled pore shape through a templating effect, that is, freeze casting or ice templating, increasing mechanical properties, and introducing anisotropic porosity.^128,312^ For instance, an aligned porous structure of alginate/chitosan cryogel with a pore size of ∼60–80 μm was produced by freeze-drying, and this architecture was used to guide the growth of neurites.^313^ If we want to maintain the porous hydrogel structure, special drying techniques are needed, yielding aerogels. Supercritical CO_2_ drying enables the removal of a solvent from a solvogel (a solvent-exchanged hydrogel, in which water is replaced with supercritical CO_2_ miscible solvents, commonly acetone or EtOH) without affecting the gel structure.^128^ Polysaccharide aerogels from supercritical drying techniques are generally of a higher specific surface area than the respective cryogels.^221,314,315^
E ***Electrospinning*** is used to create mesh-like structures with a fiber diameter in the nanometer and micrometer scales. Electrospinning uses an electric force to draw charged threads of polymer solutions. Challenges are, among others, the stability of such structure in water, especially for water-soluble polysaccharides. For instance, an electrospinning of native alginate requires a subsequent gelation step with multivalent ions, for example, Ca^2+^, Sr^2+^, or Ba^2+^ to avoid disintegration of the spun fibers.^316,317^ The electrospinning of agarose was facilitated in ionic liquids and enabled the direct fabrication of water-stable fibrous mats with antimicrobial properties.^318^
F ***Nanoparticles*** of a polysaccharide are generally produced by a controlled nanoprecipitation, which can be induced by different approaches, for example, complexation^319^ or with an antisolvent.^320^ Other production techniques are analogous to the bead formation using a microfluidic channel. Oil-in-water nanoemulsions of an alginate-chitosan mixture were prepared and subsequently gelled with Ca^2+^; simultaneously, the particles can be loaded with drugs.^321^ κ-Carrageenan composite nanoparticles were produced through complexation with the protein ovalbumin and used as a drug delivery platform.^319^ Nanoparticles can be as well obtained by controlling the biopolymer solubility through a slow addition of an antisolvent.^320^ A chemically modified alginate with a hydrophobic photosensitizer enabled the formation of nanoparticles with a hydrophobic core and hydrophilic shell.^320^ This formation was triggered by an addition of doxorubicin, forming the hydrophobic particle core, and slow solvent-exchange by exchanging a polar organic solvent with water. Algal nanocelluloses are intrinisc nanopaticles of rod-lke or nanofibrillar shape, and can be following plant nanocellulose protocols. The rheology of nanocellulose makes it especially useful for 3D printing and injectable cell delivery systems.^133,322^

## Biomedical Applications of Hydrogel-Forming Algae Polysaccharides

**Figure 6:**
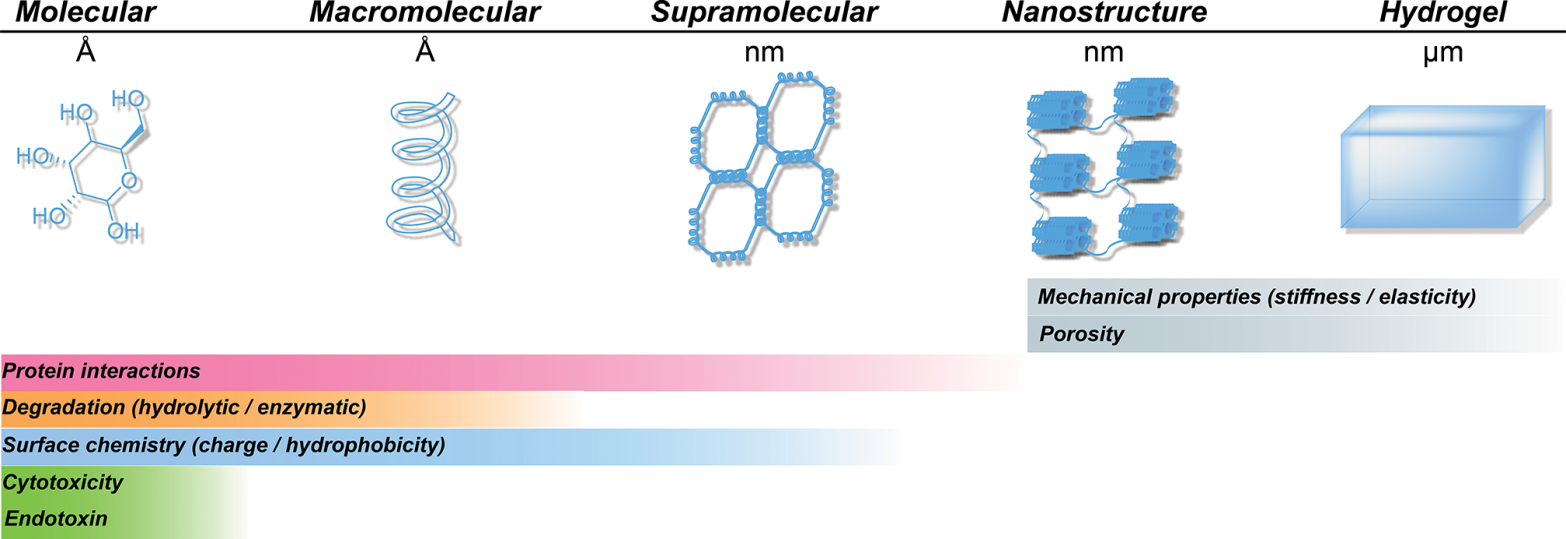
Properties of algae-extracted polysaccharides at different scales that impact the biomedical performance and interaction with biological systems.

Algae-extracted hydrogel-forming polysaccharides have an historical use in biomedical applications: agarose gel electrophoresis, agar as bacteria culture media, and alginate-based wound dressings. But with the rise of algae culture, the discovery of new polysaccharides and the development of a chemical modification of an existing polysaccharide, we expect that new biomedical applications for these materials will be considered in the future. To carefully evaluate a polysaccharide as a candidate material for a dedicated biomedical application, one must evaluate specific properties, which influence the interactions of polysaccharides with biological systems. Considering hydrogel-forming polysaccharides as a multiscale system, it is important to understand the type of interaction at each chemical and structural level (Figure 6).

First, it is important that a given polysaccharide is compatible with conventional sterilization methods. During extraction, processing, and preparation for its final use, the biomaterials might gather biological contaminants. Sterilization of the polysaccharide can be challenging, as γ-radiation and ethylene oxide treatments can induce unwanted cross-linking and depolymerization, and steam sterilization in an autoclave can cause a hydrolysis of the polysaccharide. Hence, it is crucial to carefully select a suitable sterilization method for a given polysaccharide. The discussed methods kill bacteria and viruses, but they do not remove endotoxins, which can induce a severe immune response of implanted materials leading to a fever and the rejection of the implanted materials from the body. Originating from Gram-negative bacteria, endotoxins, also called lipopolysaccharides, are macromolecules made of lipids and polysaccharides, and they can be detected by Limulus amebocyte lysate tests.^323^ Because of their chemical nature, it is extremely challenging to remove them from a polysaccharide, and usually, processes involving several acidic and basic washing steps are needed.^324^

Once a sterile material is obtained, its cytotoxicity can be characterized. One of the sources of cytotoxicity originates from impurities present in the polysaccharide. Molecules such as heavy metal or low-molecular-weight polysaccharides can be a potential source of cell toxicity. Usually, the cellular toxicity is assessed by astetrazolium-based, trypan blue, alamar blue, lactate dehydrogenase, and neutral red uptake assays.^325^

Beyond the polysaccharide purity, its chemical structure ultimately determines the interaction with biological systems. Protein adsorption plays a significant role, as it dictates the interaction of the hydrogel with living tissue and the immune system. In this regard it is important to consider not only the type of adsorbed protein but also the folded structure.^326−328^ The surface chemistry of the polysaccharide can be chemically altered to control protein adsorption. A selective introduction of cell-adhesion peptides such as the integrin binding sequence arginylglycylaspartic acid (RGD) protein enables, for example, a controlled cell attachment onto the polysaccharide.^329^ The sorption of proteins can be studied in model systems via surface plasmon resonance or microgravimetry measurements, which enable as well a subsequent studying of the protein structure by analyzing the adsorbed protein on the polysaccharide surface by AFM.^330,331^

If a hydrogel is injected, implanted, or used in the body as part of a medical device, the algae-extracted material can be in contact with blood. So its hemocompatibility—a measure of the thrombotic response of a material in contact with blood—is critical.^332^ Some functional groups of polysaccharides allow the control of its hemocompatibility. For instance, it as has been shown that sulfated polysaccharides such as carrageenan^333^ or chemically sulfated agarose can have antithrombic properties.^256^

During the lifetime of a hydrogel in the human body, the polysaccharide can degrade into oligomeric or monomeric products. These moieties can induce cytotoxic reactions or interact with proteins through electrostatics or hydrophobic interactions. As mammalian cells usually lack the suitable enzymes to break down these polysaccharides, degradation mainly occurs through an unspecific hydrolysis of the glycosidic bounds.^334,335^ Only enzymes obtained from bacteria or fungi such as agarase, alginase, or cellulase can specifically depolymerize agarose, alginate, or cellulose, respectively. Polysaccharides can be rendered more degradable by oxidative treatments, introducing defects and hydrolytically labile units in the polymer backbone.^336^

Not only the surface chemistry plays a major role in governing the polysaccharide interaction with its surrounding environment but also the topographical structure, the pore structure, and pore interconnectivity. As an example, the roughness of the hydrogel can be sensed by tissue cells, which can alter their behavior as a function of the roughness.^337,338^ In addition, to enable homogeneous in-growth and cell proliferation in a hydrogel a pore size of several 100 μm is required.^229,339^ Apart from the size, also the presence of an interconnected, open-porous hydrogel structure is viable for tissue engineering, as it allows a diffusion of nutrients, gases, and cellular waste substances.^340^

However, one must consider the influence of such a porous structure on the mechanical properties of the resulting hydrogel. In tissue engineering, the matrix stiffness and elasticity of the hydrogel should represent the targeted tissue, as cells can feel and respond to their environment.^238,341^ The human tissue elasticity varies between tens of pascals and several gigapascals from soft brain to hard bone tissue.^335^ This broad range of mechanical properties highlights the necessity of adapting the hydrogel’s mechanical properties to the targeted tissue implantation. Fine-tuning the hydrogel mechanical properties can be achieved through a chemical modification of the polysaccharide chains.

Considering this advanced knowledge on the relationship between a chemical structure of the polysaccharide and the behavior at the biological interface, algae-extracted hydrogel-forming polysaccharides possess an untapped potential in diverse biomedical applications.

## Challenges and Opportunities for Biomedical Application of Polysaccharides

Naturally occurring polymers offer several advantages over synthetic polymers, especially when it comes to a predictable property profile, for instance, gelling behavior, rheological properties, and biodegradation. While their biological origins ensure a narrow molecular weight distribution and well-conserved chemical composition, this latter trait is highly dependent on the geographical source of the polysaccharide, the extraction process, and postmodification. For example, the source of alginate can alter its composition, that is, high G versus high M content.^342^ Similar challenges are also envisioned with other marine polysaccharides such as carrageenan and agarose. But one bottleneck in the utilization and the industrial exploitation of marine algae-derived polysaccharides is the lack of physical parameters to describe the relationship between the molecular weight of these polysaccharides and their rheological properties. An empirical and semiempirical framework for understanding structure–property–function relations in macromolecules should allow for a rapid optimization of process parameters to achieve specific property profiles. In polymer science, the ‘*K*’ and ‘α’ parameters of the Mark–Houwink–Sakurada (eq 1) equation provides a basis for relating the viscosity of a polymer in a solvent at a given temperature to its molecular weight.
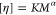
1[η] is the intrinsic viscosity (mL/g) commonly referred to as the Staudinger Index, *M* is the viscosity molecular weight (g/mol), and *K* and α are the Mark-Houwink parameters.

With the exception of alginate, one of the most well-studied polysaccharides, where *K* = 2 × 10^–3^ and α = 0.97, at 25 °C, in a solution of 0.1 M sodium chloride, such a deep characterization of marine-derived polysaccharides is virtually absent.^343^ In a biomedical application, the molecular weight (MW) and MW distribution are important considerations, as they can have unintended consequences on the biocompatibility, processing, and reproducibility of hydrogel properties. Since polysaccharide sources and extraction processes can impact the MW, a rapid and reliable way to qualify a polysaccharide and control the MW at the source will be vital to the development of marine-algae-derived polysaccharides as a reliable raw material for biomedical applications. Toward this objective, the development of analytical methods and representative polymer standards for determining molecular weights is essential. Currently, pullulan, a polysaccharide produced by a fungal action on starch, is used as a molecular weight standard. Since pullulan is not a charged polysaccharide (almost neutral), its solution behavior does not accurately represent the conformation of algae-derived polysaccharides, which are typically negatively charged. Since many of the polysaccharides such as alginate and agarose are thought to undergo degradation in vivo through oxidative processes, the molecular weight will have a pronounced effect on the solubility of oxidized polysaccharide chains, and this can influence tissue clearance and excretion and skew the biocompatibility of these polysaccharides. These challenges in establishing clear structure–property–function (both physical and biological) relationships within a polysaccharide class sourced under diverse cultivation conditions can pose regulatory and clinical translation challenges. Finally, the endotoxin burden inherent to natural polymers can be a challenge. While endotoxin removal is not a significant consideration for food technology and other consumer product applications, it is a critical factor in the clinical translation of algae-derived biomaterials and, therefore, needs to be integrated into the purification step. All these aspects of sourcing and purification must be incorporated within a comprehensive framework of characterization to allow for the processing of these polysaccharides into biomedical applications.

## Expanding the Current Polysaccharide Library and Their Applications

**Figure 7:**
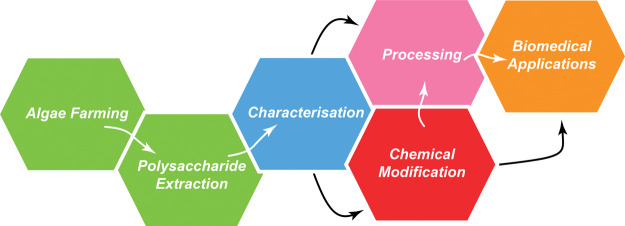
Proposed life cycle of hydrogel-forming polysaccharides derived from algae culture: their extraction, characterization, and processing into materials for biomedical application. If required a prior chemical modification can be integrated to tune their properties toward a dedicated processing and biomedical application.

The current increase of algae farming has been focusing on the potential of algae for carbon dioxide fixation, fuel production, or as a food source, but there is a real potential for algal polysaccharide hydrogels in high added-value applications in the fields of biology and medicine. One challenge in the development of such applications is the requirement of a reliable supply chain from the sea to the patient (Figure 7).

This starts with a controlled environment for the algae growth in a natural reserve or in a land culture to ensure a low contamination of the algae and to reduce seasonal variations of the polysaccharide compositions. Followed by extraction processes, which are compatible with industrial manufacturing, an efficient isolation of pure polysaccharides is enabled. This requires an appropriate quality control to characterize the algae in terms of contaminants, purity, and chemical structure. Once isolated and purified, the polysaccharide can be either directly processed into a biomaterial or chemically modified to tailor its properties before processing, thus enabling a broader application range.

**Figure 8:**
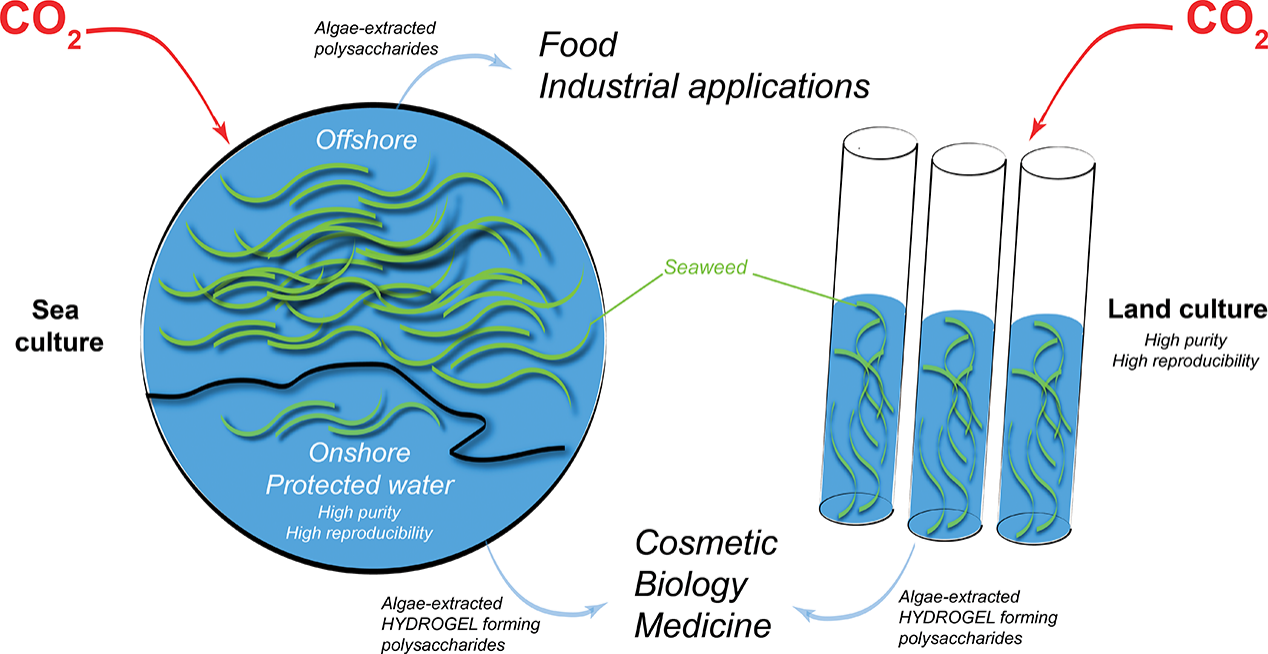
Implementation of the valorization of hydrogel-forming polysaccharides for high-value-added applications into existing supply chains for food and industrial applications from algae.

While animal-derived biomaterials, such as collagen, remain the main source of protein-based hydrogels, algal hydrogel-forming polysaccharides represent a viable, nonanimal alternative and can be efficiently processed into high added-value biomaterials with low impurity levels and batch-to-batch reproducibility. Nowadays, there are established processes and an available industrial supply chain to expend the library of hydrogel-forming polysaccharides from algae. Research on new algae species and the development of novel extraction processes could further support the growth of this industry by opening new opportunities beyond the well-established ones of alginate and agarose. A high potential for valorization (Figure 8) could be found in the cellulose content in algae. Indeed, algal cellulose can be further processed into high-purity nanocelluloses,^148^ which can be used, for example, as an artificial extracellular matrix and rheology modifier in bioinks.^12,344^

Parallel with the development of an algae industry for carbon dioxide fixation, additional uses of the produced algae will have to be found. While mass markets such as algae-based industrial chemicals and food will be a major part of the equation, these require high production capabilities. Smaller algae producers with access to high-quality water will have the opportunity to produce materials to support the high added-value chain of biomaterials. This industry, however, requires the production of high-purity polysaccharides in a reproducible composition and with low seasonal variations between harvests. This can be achieved with the development of an advanced algae culture, such as an inland production, which can lead to a high-quality polysaccharide. In addition to high reproducibility, an inland production allows extensions of algae culture to many areas with no or limited sea access (Figure 7).^345^

Taking this into account, the market of hydrogel-forming polysaccharides from algae has the potential to grow and develop further in different regimes. An ongoing important task is the proceeding research on new and optimized extraction methods, which can allow us to extract polysaccharides with superior properties, for example, polysaccharides with a higher molar mass or in higher purity, or to increase further the extraction yields. Moreover, because of the huge varieties of algae species, many types of polysaccharides fractions with yet unknown compositions and properties are still available with undeveloped potential. Currently, algal polysaccharides are primarily exploited on their own, although they have strong synergistic effects in the natural algae matrix; these properties can be further investigated to obtain materials with hitherto unattainable mechanical properties. Supported by the current knowledge in chemistry and material science of algal hydrogel-forming polysaccharides, this growing industry is expected to further advance and lead to the development of new biomedical applications.
